# Recent Advances in Bitterness-Sensing Systems

**DOI:** 10.3390/bios13040414

**Published:** 2023-03-23

**Authors:** Yanqi Li, Nigel Langley, Jiantao Zhang

**Affiliations:** 1 Cixi Institute of Biomedical Engineering, Ningbo Institute of Materials Technology and Engineering, Chinese Academy of Sciences, Ningbo 315201, China; 2 Gaylord Chemical Company LLC, 1404 Greengate Dr, Ste 100, Covington, LA 70433, USA

**Keywords:** bitterness, taste sensors, bitterness evaluation, electronic tongue, taste masking

## Abstract

Bitterness is one of the basic tastes, and sensing bitterness plays a significant role in mammals recognizing toxic substances. The bitter taste of food and oral medicines may decrease consumer compliance. As a result, many efforts have been made to mask or decrease the bitterness in food and oral pharmaceutical products. The detection of bitterness is critical to evaluate how successful the taste-masking technology is, and many novel taste-sensing systems have been developed on the basis of various interaction mechanisms. In this review, we summarize the progress of bitterness response mechanisms and the development of novel sensors in detecting bitterness ranging from commercial electronic devices based on modified electrodes to micro-type sensors functionalized with taste cells, polymeric membranes, and other materials in the last two decades. The challenges and potential solutions to improve the taste sensor quality are also discussed.

## 1. Introduction

Mammals have the following five basic taste sensations: sweet, bitter, sour, umami, and salty. These five sensations play a significant role in animals and human beings distinguishing nutrition and toxins [1,2]. Among the five basic tastes, the bitter taste is special as it arouses unpleasant emotional responses that keep mammals away from toxins and perished foods, protecting their health from being affected by poisons [3,4,5]. Although medicines and food products are beneficial to health, their bitter taste in some cases brings unpleasant sensations and may cause a negative impact on consumer compliance and acceptance [6,7,8,9,10]. To address these problems, scientists have developed many taste-masking methods to efficiently inhibit or block bitterness, e.g., using flavors and sweeteners, microencapsulation, hot melt extrusion, coatings, and nanohybrid technologies [11,12]. Bitterness detection has become critically important in evaluating the taste-masking method’s effectiveness and screening and optimizing formulations in the pharmaceutical and food industries.

**Figure 1:**
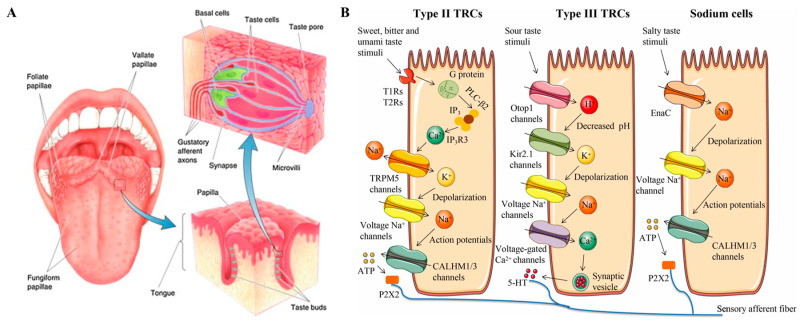
Schematic structure and taste transduction of the human taste-sensing system. (**A**) The distribution and structure of different papillae, taste buds, and taste cells of the tongue; (**B**) diagram of taste signal transduction in different types of taste cells responding to bitter, umami, sweet, sour, and salty compounds. Reproduced from [13,16] with permission from Elsevier.

In the past decades, many investigations have been published that have helped provide a deep understanding of the mammalian gustatory system. The taste transduction process includes two steps: perception and coding [13]. For mammals, many circumvallate papillae, foliate papillae, and fungiform papillae are distributed on different areas of the tongue, and each of them contains dozens to hundreds of taste buds [14]. Every taste bud is composed of several taste cells which are connected to corresponding nerve fibers [15]. The process of taste perception happens when the taste cells make contact with the bitter actives in foods and medicines, and then the cellular signals are coded and transmitted to the relevant brain structures (Figure 1) [16].

According to ultrastructural features, taste cells can be classified into type I, type II, type III, and type IV [17]. Each type of taste cell expresses one or more taste receptors [17,18]. Among the five basic taste sensations, the sweet, umami, and bitter tastants are recognized by G-protein-coupled receptors (GPCRs) which are mainly expressed on type I and type II taste cells [19,20]. The bitter taste receptors contain over 25 members that can detect bitter compounds and are classified as the TR2 family [21,22,23,24]. Many studies have clearly explained the taste transduction mechanism in bitter taste cells (see Figure 1B) [25,26,27,28]. Briefly, the bitter taste perception of cellular changes is produced by participating transient receptor potential channels (TRP), phospholipase, and organelles, simultaneously releasing adenosine triphosphate (ATP) and neurotransmitters for neural signal transmission [29,30,31,32]. Additionally, bitter taste receptors have also been found in cells of the digestive system, respiratory system, brain, and testes [33,34,35].

The bitterness assessment has in vivo and in vitro approaches [11]. Traditional in-vivo evaluation contains a human test panel [36,37] and an animal ethology test [38,39]. In the human test panel, bitterness evaluation is carried out by a group of pretrained volunteers with standard taste protocols. This method can directly provide the bitterness assessment results, but it is constrained by the volunteer experience level, individual differences, time consumption, and toxicity risks [30]. Rats are commonly employed for bitter taste detection in the animal test by comparing their preference for test products. This approach is economical and time-efficient, in addition to omitting toxicity risks [40]. The bitterness test results sometimes cannot represent the real bitterness of the samples due to the interspecies difference between animals and human beings. Thus, there is a demand to develop fast, safe, effective, and cheap in vitro methods for bitterness evaluation. In recent decades, scientists have published a large number of new methods using instruments for in vitro bitterness detection, including taste strips [41], facial expression analysis [42], and bitter taste sensors [43,44]. Here, we summarize the recent progress in the development of novel taste sensors for bitterness detection, including commercial electronic devices based on modified electrodes to micro-type sensors functionalized with taste cells, polymeric membranes, and other materials. The principle of sensor construction, material selection, and utilization of these bitter taste-sensing systems in taste-masking assessment are discussed. Lastly, the future perspectives for bitter taste sensor improvement are considered.

## 2. Electronic Tongues in Bitterness Evaluation

**Figure 2:**
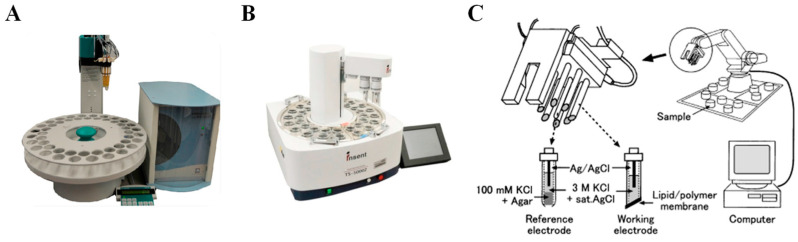
Commercially available E-tongues: (**A**) Alpha M.O.S E-tongue Astree II; (**B**) Insent E-tongue TS-5000Z; (**C**) Insent E-tongue SA402B. Reproduced from [55,57] with permission from Elsevier.

Inspired by the mammalian gustatory system; taste sensing systems based on arrays of modified cross-sensitive electronic sensors were designed for in vitro bitterness evaluation, called electronic tongues (E-tongues) [45,46,47]. Cross-sensitive electrodes modified by special membranes which consist of lipids/polymers or organic coated materials can respond with global selectivity to the five basic taste objectives [48]. Thus, E-tongues can identify and distinguish five basic tastes at the same time. Typically, the selectivity of the working electrodes can be regulated by changing the categories and concentrations of the lipids, polymers, and other materials used [49,50,51]. Electrochemical measurements include potentiometry, amperometry, voltammetry, and impedance spectroscopy [52,53,54]. Among these techniques, potentiometry is the most widely utilized. Currently, three commercialized E-tongues based on the potentiometry sensing mechanism are marketed and employed in various applications, branded as the Astree II (Alpha M.O.S, France) [55], TS-5000Z [56], and SA402B [57] E-tongues (Insent Inc., Atsugishi, Japan) tongues (Figure 2).

### 2.1. Astree II E-Tongue (Alpha M.O.S.)

The Astree II E-tongue system consists of a detection sensor set, an electronic unit, and multivariate statistical analysis software [58]. The detection sensor set has six special electrodes which are coated with different proprietary organic membranes (ZZ, AB, BB, CA, DA, and JE) [59]. Additionally, one Ag/AgCl reference electrode and one stirring rod contained in the detection set can help measure the voltage difference between each working electrode and the reference electrode. On the bottom of each special sensor, the coated organic membrane and an ion-selective field-effect transistor (ISFET) are integrated into a microchip. The selectivity and sensitivity of these sensors are governed by the coating materials [60]. After binding with the dissolved ions and taste substances, the chemical-sensitive membrane generates a change in membrane potential, which is measured and then analyzed by a multivariate statistical analysis method, such as principal component analysis (PCA) [61]. The Astree II E-tongue has many advantages, such as high repeatability, robustness, acceptable accuracy, and short detection time (within 2 min), making it widely used for bitterness evaluation in the pharmaceutical industry.

Many studies on the application of the Astree II E-tongue for bitter taste detection have been reported [62,63,64]. For instance, Li and coworkers used the Astree II E-tongue to measure the bitterness intensity of 35 different traditional Chinese medicines [65]. The bitterness measurement of the Astree II E-tongue was optimized with standard bitter substances. Additionally, the E-tongue was successful in bitterness detection with good reproducibility and performed high precision in a 6 h measurement cycle. The least squares support vector machine (LS-SVM) was utilized for analyzing the bitterness data of traditional Chinese medicines, which achieved a binary classification accuracy of 100% and a ternary classification accuracy of 89.66%, respectively. Furthermore, the Astree II E-tongue has been employed for assessing taste-masking effectiveness and optimizing pharmaceutical formulations on the basis of its discrimination ability [66,67,68,69,70,71,72,73]. Wang and colleagues used the Astree II E-tongue to design and select the additives of levetiracetam instant-dissolving tablets made by 3D printing [74]. The bitterness of the tablets with different additives was compared to that of the placebo samples (without a pharmaceutical active) in different concentrations. PCA helped screen the satisfactory additives and the suitable concentrations, which provided consistent results with the trend from the human test panel.

### 2.2. SA402B and TS-5000Z E-Tongues (Insent Inc.)

Unlike the Astree II E-tongue, the SA402B and TS-5000Z E-tongues combine various working electrodes modified with different types of lipid/polymer membranes [75,76]. The key technology of these E-tongues is the lipid/polymer membrane on electrodes, which comprises a lipid, a plasticizer, and a polymer as the sensing part to receive taste substances [77]. Each measurement cycle is carried out by recording a reference solution (V_r_), a sample solution (V_s_), and the aftertaste solution (V_r_’), followed by a cleaning process. The difference between V_s_ and V_r_ is identified as a relative value, while the difference between V_r_ and V_r_’ is used to assess the change of the membrane potential caused by molecular adsorption [59,78]. The selectivity and response intensity of the lipid/polymer membrane to taste objectives depend on the hydrophobicity and ionization capacity of the lipids, and they can be adjusted by replacing different lipids in the membrane or changing the lipid concentration [79,80,81]. Specifically, the membrane on the bitterness-sensing electrode has a lower amount of charged lipid in the membrane than in the salty-sensing electrode, making the bitterness electrode membrane more hydrophobic and allowing bitter compounds to be more easily adsorbed onto the membrane. Therefore, the changes in the sensor membrane potentials vary, giving E-tongues the ability to distinguish the five basic tastes. Thus, SA402B and TS-5000Z have been widely used for pharmaceutical and food product bitterness detection [82,83,84,85,86,87,88].

Liu et al. used SA402B to characterize Chinese mitten crab meat from three groups: the native culture area in Yangcheng Lake, Yangcheng Lake-labeled crabs from the market, and aquaculture ponds [89]. The E-tongue data showed that umami and sweetness were the main tastes in crab meat, while saltiness and bitterness varied in crabs from different geographic origins. PCA and linear discriminant analysis (LDA) verified that the SA402B system could classify those crabs obtained from different origins with an accuracy of 100%, which provided an efficient method to monitor the food quality. Sayuko and other researchers utilized the SA402B taste system to evaluate the suppression of chlorogenic acid (CGA), which is an ester formed between caffeic acid and quinic acid for the synthesis of bitter basic drug diphenhydramine hydrochloride (DPH) [90]. The bitterness intensity was predicted by the membrane potential change caused by adsorption of the bitter drug. The bitterness of DPH was suppressed by CGA in a dose-dependent manner. Consistent with the results obtained by the human gustatory sensation test, the electronic taste system could identify the bitterness intensity of DPH and the taste-masking effectiveness. With ^1^H-NMR analysis, the researchers found that the electrostatic interaction between the carboxyl group of chlorogenic acid and the amine group of diphenhydramine hydrochloride prevented the membrane adsorption of DPH molecules, which efficiently reduced the bitterness intensity. Huang and colleagues succeeded in selecting naringin dihydrochalcone and neodiosmin from 12 citrus flavonoids for the bitterness inhibition of naringin, quinine hydrochloride, and stevioside with the SA402B system [91].

The TS-5000Z taste system also displayed good performance in bitter taste detection and quantitative prediction of bitterness masking. As an example, Wu and coauthors utilized the TS-5000Z to evaluate the bitterness suppression effect of high-potency sweeteners such as aspartame and saccharine sodium on quinine hydrochloride [92]. On the basis of a regression analysis model obtained from the bitterness sensor and the sweetness sensor, scientists proposed bitterness prediction formulas to describe the bitterness perception balanced by sweetness in the human brain to predict bitterness intensity after taste masking. The perception results showed a good correlation with the human test panel results. In another study, Alison and colleagues used TS-5000Z equipped with four bitter sensor electrodes to evaluate the bitterness-masking efficiency of the formulations constituting isoniazid with Soluplus and Eudragit E-PO via a hot melt extrusion process [93]. The bitterness intensity of several formulations recorded by the taste system was consistent with the bitter taste suppression of the two polymers for the drug by detecting taste-extracted water. The Euclidean distance between pure drug and taste-masked formulations in the PCA map demonstrated the taste difference, providing indications to optimize the proportion of masking materials and to accelerate the formulation screening process.

It was demonstrated by Katharina and coauthors that the Atsree II system has similar properties to the Insent taste sensing system when sensing bitter ionic and neutral drug substances [48]. Both systems showed good reproducibility in bitterness evaluation, and the results could be correlated to those from the human test panel. In addition to the inter-day detection, the result provided by the Insent taste sensing systems was normalized with a standard solution after each measurement, while all data obtained by the Astree II system needed to be normalized before comparison.

### 2.3. Other E-Tongues

In addition to commercialized instruments, some other taste-sensing systems have also been assembled in the laboratory and utilized in bitterness detection. For example, potentiometric electronic systems based on a combination of various functional lipid/polymer membrane electrodes have been constructed and applied for bitterness evaluation [94,95,96]. For these instruments, the researchers changed the lipids used in membranes or increased the number of electrodes to record more detailed information about bitter substances, which could improve the discrimination ability of the taste-sensing systems. All-solid-state electrodes (ASSEs) were modified with a conducting polymer and used to fabricate potentiometric taste sensors. Ewa and Maria designed a taste sensor armed with five ASSEs (ASSE III) and succeeded in developing two prediction models of the concentration of quinine hydrochloride and total saccharides in seven tonic water commodities through multivariate linear regression [97]. In another study, Janine et al. accomplished the bitterness evaluation of artesunate-mefloquine (ASMQ), praziquantel (PZQ), and benznidazole (BNZ), using a homemade impedimetric E-tongue [98] that was constructed with an array of five interdigitated gold electrodes modified with layer-by-layer films that were deposited by alternatively immersing the electrodes in cationic and anionic polymer solutions. The recorded bitter taste intensity of these drugs was analyzed using a multidimensional projection technique (the Interactive Document Mapping, IDMAP), and the results showed large differences compared to the placebos, indicating that the taste-sensing system could be a promising method for bitter drug detection.

## 3. Microminiaturized Biosensors for Bitterness Detection

Along with recent advances in studying receptors and cells in mammalian taste systems, many biological materials, such as taste tissues [99,100], tongue buds [101], cells [102], and bitter receptors [103,104], have been selected to construct novel E-tongues, which are also called “BioETs” [105,106]. To record the change in electronic signals, many basic micro-electronic components have been used as transducers for bitterness taste biosensor construction: the quartz crystal microbalance (QCM) [107], microelectrode array (MEA) [108], light-addressable potentiometric sensor (LAPS) [109], cell-based impedance sensor (CIS) [110], and others [111,112].

### 3.1. Animal Gustatory Cortex

**Figure 3:**
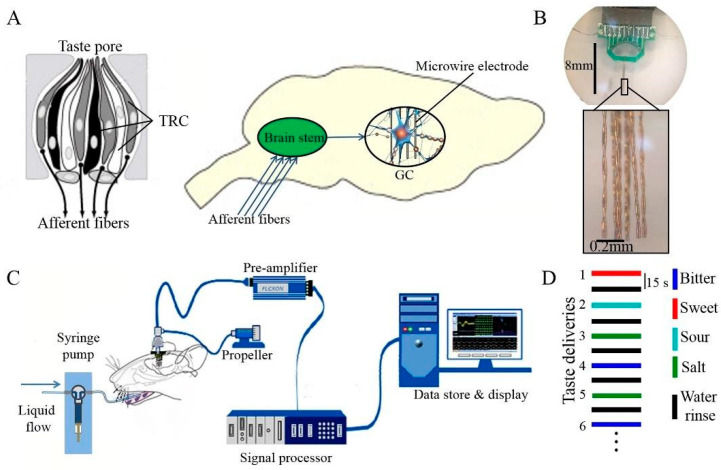
Bitterness biosensor constructed with rat’s gustatory cortex. (**A**) Scheme of a rat gustatory system and the taste information pathway from taste buds via brain stem to microwire electrodes; (**B**) the microelectrode array employed in biosensor; (**C**) the schematic diagram of the whole animal-based biosensor; (**D**) the test order of different taste deliveries. Reproduced from [114] with permission from Elsevier.

Biosensors based on animal mammalian gustatory systems provide a whole sensing strategy for bitterness detection without requiring complex microelectronic array modification [113]. For instance, Qin and colleagues constructed a biosensor using a rat mammalian gustatory system for bitterness detection (Figure 3) [114]. After brain surgery, a 16-channel microelectronic array was inserted to anesthetic rats’ dura and stabilized with a dental acrylic resin. By recording the extracellular potentials, the response to bitterness could be recorded within 4 s. PCA was utilized in data analysis, and the response patterns of these five tastes were easily distinguished with an accuracy of 94.05%, compared to commercial E-tongues. This in vivo system was stable enough to detect the bitterness for at least 1 month. Although these biosensors that were built with whole animal gustatory systems directly provided the straightforward bitterness detection result in the shortest response time, the complex fabrication process, strict rat-feeding requirement, and professional surgery operations could not eliminate the errors among different rats, which limits the wide application of animal-based biosensors in the industry.

### 3.2. Bitter Taste Receptors

To overcome the limitations in animal-based biosensors, human bitterness receptors with high selectivity to bitter substances have attracted researchers’ attention. Generally, these receptors can be immobilized on suitable transducers, and the sensor size can be minimized. Thus, they not only respond to bitter substances with satisfactory selectivity, but also possess a shortened response time.

In 2012, Song et al. first reported a novel bitter nanobioelectronic tongue modified by a human bitterness receptor, hTAS2R38 [115]. In addition to human taste cells, hTAS2R38 is also expressed at a high level in Escherichia coli. It enables specific recognition and perception of bitter compounds containing thiourea (N−C=S) moieties, such as phenylthiocarbamide (PTC), propylthiouracil (PROP), and antithyroid toxin. The bitterness receptor was first immobilized on carboxylated polypyrrole nanotubes (CPNTs), and then the receptor conjugated CPNT was combined with a field effect transistor (FET) for recording the electrical signal changes. Compared with a non-taster-type receptor hTAS2R38, the fabricated nanobioelectronic sensor showed a high sensitivity and selectivity in bitter detection of PTC, PROP, and antithyroid toxin. The detection limit could reach as low as femtomolar for these bitter analytes, even after being mixed with sweeteners, umami tastants, and other bitter compounds. The taste sensor was further utilized in detecting these species in vegetable samples. The results verified it is a fast and labor-saving bitter detection biosensor with the possibility of use in real applications. Another human bitter receptor, hT2R4, was also found efficiently expressed in E. coli. Wang and colleagues developed a new biosensor by utilizing an indium tin oxide (ITO)-based electrolyte–semiconductor (ES) structure as the electrical transducer [116]. After E. coli was contacted bitter substances, the extracellular acidification of E. coli increased, which induced a potential change on the ITO-based bioelectronic taste sensor. This taste sensor exhibited a good performance in detecting the bitter compound, denatonium, with detection in 15 min at a high sensitivity of 50 nM–500 nM and a unique specificity to bitterness.

**Figure 4:**
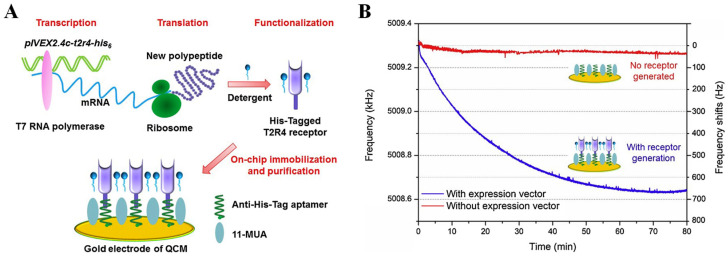
Biosensor constructed by modifying bitter taste receptors on QCM device. (**A**) Schematic design of in situ synthesis and purification steps of bitter taste receptors on QCM electrode; (**B**) the kinetics of bitter receptor proteins immobilization on the surface of QCM device via recording real-time crystal resonant frequency shifts. Reproduced from [117] with permission from Elsevier.

Although these two bitter taste biosensors satisfied the requirements in bitter taste detection, the receptor preparation and bacterial culture in traditional methods were time-consuming and complex, which limited their fabrication efficiency. Thus, Du and other researchers developed a new approach to address these disadvantages by synthesizing and purifying T2R4 bitterness receptors in situ on the QCM transducer, as shown in Figure 4 [117]. An expression vector pIVEX2.4c-t2r4-his6 containing full-length cDNA of T2R4 and a His6-tag sequence was used as the template for the bitter taste receptor in situ synthesis that was immobilized with the anti-His6 aptamers on the gold surface of the QCM device. Then, the synthesized bitter receptors were directly purified, and the whole fabrication process was finished within a few hours. The binding of the bitter substance, denatonium, was recorded by the crystal resonant frequency shifts from the mass-sensitive QCM. This bitter taste biosensor achieved threefold sensitivity enhancement.

### 3.3. Bitter Taste Cells

**Figure 5:**
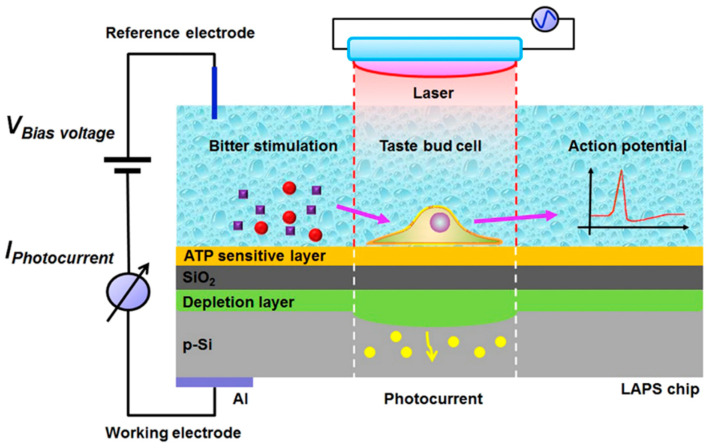
Schematic of the bitterness-sensing mechanism of the dual-functional bitter taste biosensor modified with taste bud cells and ATP-sensitive DNA aptamer on a semiconductor LAPS chip. Reproduced from [126] with permission from Elsevier.

In recent years, some new bitter taste transduction mechanisms have been reported and significantly promoted for cell-based taste sensor development [118,119,120]. Among them, a calcium-induced release mechanism has attracted most interest. When bitter substances first bind to bitter receptors on cell membranes, the heterotrimeric G protein is activated and triggers the release of two intracellular messengers, resulting in an increase in the intracellular Ca^2+^ concentration, followed by a depolarization of the plasma membrane to generate action potentials [121]. Inspired by this mechanism, researchers have built a series of bitter taste biosensors with different cell lines. In addition to taste bud cells from human tongues, many other cells expressing bitter taste receptors, such as HEK-293 cells [122], human Caco-2 cells [123], rat germ cells [124], and rat cardiomyocytes [125], have been considered as the sensing elements to improve the taste discrimination ability of bitterness biosensors. For instance, Du et al. fabricated a bitter taste biosensor by culturing taste bud cells on a LAPS, which was a photoelectric semiconductor and sensitive to surface potential changes, and then achieved the bitter detection based on a single taste cell [126]. Meanwhile, according to the bitterness-sensing mechanism of a taste bud cell where the ATP was released from cells with accompanying Ca^2+^ release, these researchers modified the LAPS surface with an ATP-sensitive DNA aptamer to measure the ATP release from the cells simultaneously, endowing the biosensor with the ability to record signal changes in both discrete and continuous modes (as shown in Figure 5). Upon adding the bitter denatonium, the cell membrane potential changes were coupled to the bias voltage supplied on the LAPS and then generated a corresponding fluctuation photocurrent. Additionally, the released ATP from illuminated taste cells could bind with the DNA aptamer on the surface of LAPS, which caused the surface charge changes and the modulation of the flat-band potential. This biosensor provided the possibility to record the taste signal transduction at the single-cell level.

**Figure 6:**
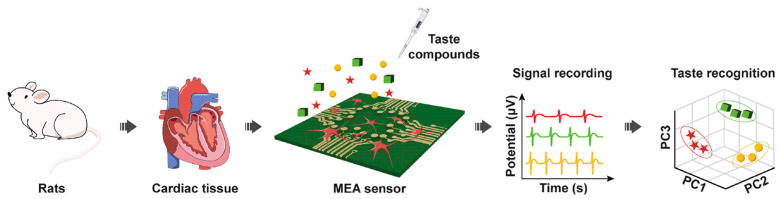
Schematic diagram of biosensor construction and bitter taste detection with rat cardiomyocytes on an MEA sensor chip. Reproduced from [109] with permission from Elsevier.

**Figure 7:**
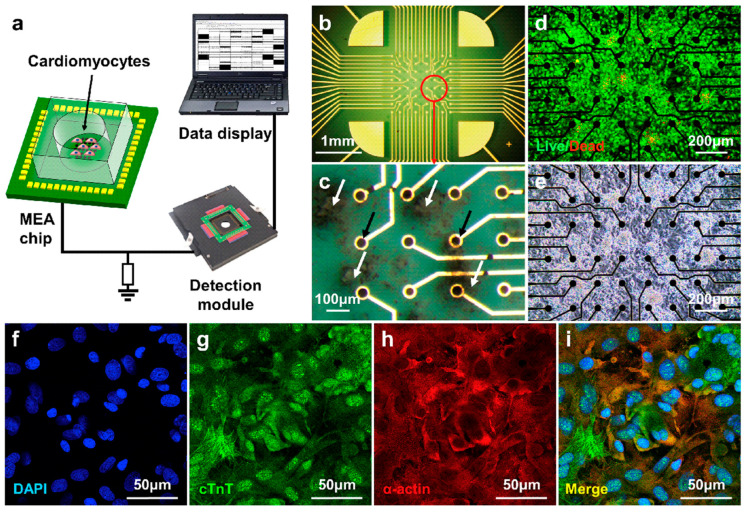
The cell culture results of cardiomyocytes on the MEA sensor. (**a**) Schematic diagram of the taste sensor system based on MEA sensor; (**b**–**e**) staining and optical micrographs of cardiomyocytes on MEA sensor; (**f**–**i**) immunofluorescence staining results for the nuclei (DAPI, blue), cardiac troponin T (cTnT, green), and α-actinin (red) in cardiomyocytes and the merged image. Reproduced from [108] with permission from Elsevier.

In another study, rat cardiomyocytes were employed as a bitter taste-sensing element by Wang’s group for the first time to functionalize the MEA transducer and construct a biosensor with simpler operations and stabler electric signals (Figure 6) [108]. In addition to bitter taste receptors, some umami taste receptors were also endogenously expressed in cardiomyocytes, thus endowing the biosensor with the ability to discriminate bitter substances (denatonium benzoate and diphenidol) and umami compounds by PCA. The immunofluorescence staining results of cardiomyocytes cultured on MEA sensors demonstrated that the cells attached and grew on the surface of an MEA chip (Figure 7). After coming into contact with different tastants, eight parameters including firing rate (FR), field potential amplitude (FPA), field potential duration (FPD), peak time, 50% rising time, 50% recovery time, second peak time, and interval time were selected to evaluate the selectivity and sensitivity of the biosensors for bitter and umami taste detection. Due to the specificity of taste receptors in cells, the taste sensor systems showed significant responses to bitter and umami compounds, but no signal changes to sweet, sour, and salty tastants. Moreover, the detection limits of denatonium benzoate, diphenidol, and monosodium glutamate were 3.46 × 10^−6^ M, 2.92 × 10^−6^ M, and 1.61 × 10^−6^ M, respectively, which was comparable to other cell-based bitter taste sensors and ensured the capability of detecting real samples.

**Figure 8:**
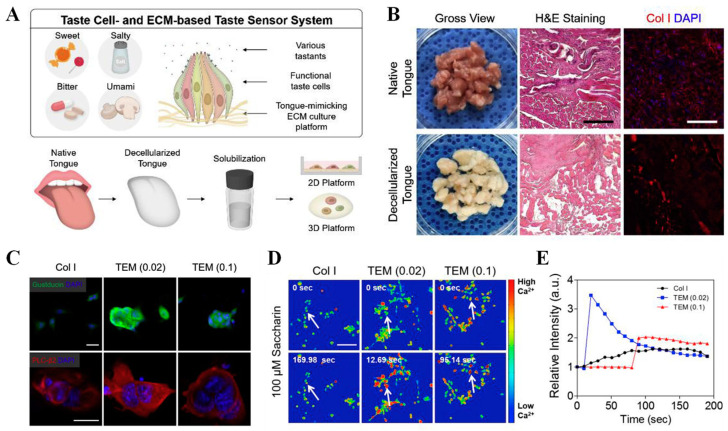
Preparation and sensing performance evaluation of TEM-based artificial tongue. (**A**) Schematic image of taste cell- and TEM-based taste sensing systems; (**B**) characterization of tongue tissue matrix before and after decellularization; (**C**) immunostaining of taste cell-specific markers, gustducin (green) and PLC-β2 (red), on Col. I-based and TEM-coated 2D platform; (**D**) changes in calcium ion flux imaging of taste cells after treating with bitter compound saccharin; (**E**) imaging analysis of taste cells’ sensing performance. Reproduced from [129] with permission from Elsevier.

Recently, some cell coculture sensors for bitterness evaluation have been reported. By coculturing Caco-2 cells and SH-SY5Y cells, which express different bitter taste receptors in various proportions, Qin and coworkers fabricated a bitterness biosensor on CIS and efficiently improved the discrimination ability for three bitter substances [127]. Furthermore, Yun and other colleagues simulated the intercellular signal communication in a bitter gustation and assembled taste cells and neuronal cells through DNA–lipid conjunctions to detect bitter compounds [128]. Through a calcium-induced release mechanism, the released Ca^2+^ concentrations in both cells were the key messengers to signal transduction. Thus, bitter substance detection could be achieved by measuring the sequential influx of calcium ions from the taste cell cytoplasm to neuronal cells through a fluorescence microscope with a fluorescent probe, Fluo-4 AM. The cell coculture sensor further provided a new approach for cell-based biosensor construction. To overcome the limitation of stable culturing techniques for taste cells, Cho’s group coated polystyrene cell culture plates with a decellularized tongue extracellular matrix (TEM) and formed two-dimensional and three-dimensional platforms with taste cells and neurons to prepare bioartificial sensing systems [129]. Benefiting from mimicking the tongue’s microenvironment, the 2D coatings and 3D hydrogel platforms ensured the functional taste cell-specific phenotypes and significantly improved the adhesion and sensitivity of taste cells to different tastants (sweet, bitter, salty, sour, and umami) over the traditional collagen type I (Col. I)-based platforms (Figure 8). By coculturing primary taste cells and mouse neurons, the response of the taste cells to different compounds could be visually observed by calcium ion influx imaging. The TEM-based artificial tongues successfully sensed coffee (30 mg/mL) and diluted wine samples (1 *v/v*%), showing a short response time and significant changes in calcium influx intensity.

### 3.4. Other Micro-Miniaturized Bitter Taste Sensors

**Figure 9:**
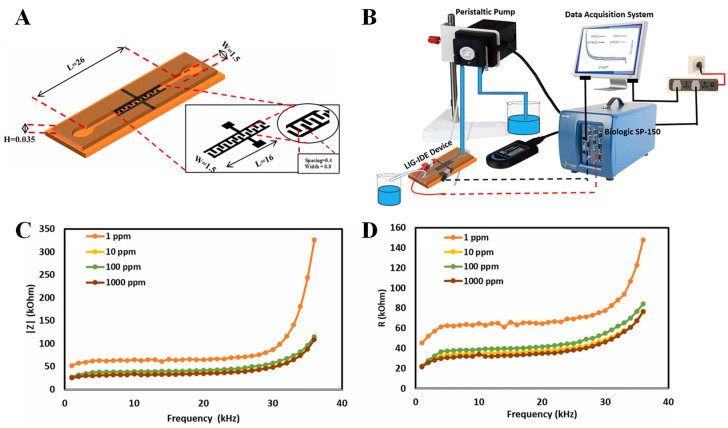
Schematic diagrams and sensing performance evaluation of laser-induced graphene-based microfluidic taste sensor. (**A**) Schematic image of microchannel and interdigitated electrodes in sensor; (**B**) diagram of complete detection system; (**C**) impedance spectroscopy results of taste sensor to L-tryptophan (bitter substance) expressed in terms of reactance on the frequency range; (**D**) impedance spectroscopy results of taste sensor to L-tryptophan (bitter substance) expressed in terms of resistance for the frequency range. Reproduced from [138] with permission of Elsevier.

In addition to the bitter taste biosensors mentioned above, some micro-miniaturized sensors have been reported. The materials used to modify the electrode elements in taste sensors include conducting polypyrrole, polymeric hydrogels, phthalocyanine complexes, carbon nanotubes, laser-induced graphene, and metal-oxide semiconductors [130,131,132,133,134,135,136,137]. Mrunali and colleagues prepared a microfluidic device with an integrated microchannel and interdigitated electrodes (IDEs) through a laser-induced graphene technique on a polymer chip, and they succeeded in discriminating five basic taste chemicals (Figure 9A,B) [138]. By monitoring the change in impedance difference between deionized water and bitter sample solutions, electrochemical impedance spectroscopy gave fast and sensitive signals for L-tryptophan at different concentrations (Figure 9C,D), which provided a fast, low-cost, and simple-to-operate bitterness-sensing method.

## 4. Conclusions

Bitterness is one of the five basic tastes and plays a significant role in mammals’ lives by preventing them from consuming toxins or perished foods. The bitterness of foods and oral pharmaceutical products arouses unpleasant sensations and feelings that dramatically impact consumer compliance and acceptance. Thus, multiple in vivo and in vitro evaluation methods have been developed to detect bitter compounds, including electronic instruments based on arrays of functional electrodes and micro-miniaturized taste sensors based on bitterness-sensitive biomedical and organic materials, as summarized in Table 1.

As for E-tongues, the advantages of commercialization, reproducibility, stability, and high-throughput detection make them a preferred choice for laboratories and companies, but they also require trained personnel to manipulate the instruments. Micro-miniaturized taste sensors, based on biomaterials, remarkably reduce the size of taste sensor systems and have better sensitivity and selectivity toward bitter substances. However, the lifetime of these biosensors is restricted by the biomaterials’ instability, and the operation procedures are complex. Thus, there is a need to understand new interaction mechanisms between bitter compounds and signal transducers, on the basis of which new taste sensing systems with long service lifetime, satisfactory stability, and low cost can be designed and developed in the future.

## 5. Perspectives

In addition to their wide use in electrical signal monitoring, optical sensors may potentially be more practical and economical for visually detecting bitter actives in food and pharmaceutical industries [139]. Color or light wavelength changes induced by the interaction between bitter tastants and sensors can be easily captured with scanners, cameras, and even naked eyes, and the data can be analyzed using RGB, CMYK, or gray value model software. This strategy may provide new solutions for the construction of biosensors able to perform low-cost and high-throughput bitterness evaluation.

## Figures and Tables

**Table 1:** Comparison of bitterness-sensing systems based on modified electrodes and biomaterials.

Bitterness-Sensing System Types	Reference	Bitterness-Sensing System	Sensing Elements	Detectable Bitter Molecules	Detection Concentrations	Advantages	Disadvantages
E-tongues	[65]	Astree II E-tongue	Electrodes modified with microchips of different proprietary organic membranes and ion-selective field-effect transistors (ISFETs)	Berberine hydrochloride, rhynchophylline, leonurine, matrine, and quinine	0.5 mM	Good reproducibility and high precision, high-throughput bitterness evaluation for large samples, and easy instrument operation	Complex calibration process before detection, long detection time, and large sample volume (>80 mL)
	[74]			Levetiracetam	10, 16.7, 33.3 mg/mL		
	[89]	SA402B E-tongue	Working electrodes modified with different types of lipid/polymer membranes	Chinese mitten crabs and quinine hydrochloride	0.04 mg/mL	Good reproducibility and high precision, high-throughput bitterness evaluation for large samples, and normalization of results to a standard solution after each measurement	Complex calibration process before detection, long detection time, and large sample volume (>80 mL)
	[90]			Diphenhydramine hydrocholoride	0.5 mM		
	[91]			Naringin, quinine hydrochloride, and stevioside	0.48 g/L 0.1 g/L 0.25 g/L		
	[92]	TS-5000Z E-tongue		Quinine hydrochloride	0.1 mM		
	[93]			Isoniazid	10 mg/mL		
	[94]			Sodium saccharin, ibuprofen lysinate, ibuprofen, acetaminophen, caffeine, caffeine citrate, and quinine hydrochloride	1 mM 0.013 mM 0.013 mM 0.013 mM 0.05 mM 0.03 mM 0.02 mM		
	[98]	Other E-tongues	All-solid-state electrode (ASSE) modified with a conducting polymer	7 tonic waters (containing quinine hydrochloride)	/	Global selectivity for quantitative and qualitative analysis, satisfied stability, sensitivity and reproducibility	Complex fabrication process
	[99]		Interdigitated gold electrodes modified with layer-by-layer films of cationic and anionic polymer	Artesunate-mefloquine, praziquantel, and benznidazole	0.75 mg/mL 1.5 g/mL 0.125 mg/mL	Good sensitivity, correlation of detection results with in vivo results, and evaluation of neutral bitter substance	Complex fabrication process of electrodes
Biosensors	[115]	Animal gustatory cortex with inserted 16-channel microelectronic array		Denatonium benzoate	Lower than 0.1 µM	Fast (within 4 s) and stable (1 month) response, straightforward bitterness detection, and high accuracy	Complex fabrication process, strict rat-feeding requirement, and professional surgery operation; difference in bitterness response between human and rats cannot be eliminated
	[116]	Combined human bitterness receptor hTAS2R38 with immobilized carboxylated polypyrrole nanotubes on a field-effect transistor		Phenylthiocarbamide (PTC), propylthiouracil (PROP), goitrin, and allylisothiocyanate	1 fM 10 fM 100 pM 1 nM	High sensitivity and selectivity for bitterness; time and labor-saving	Complex fabrication process
	[117]	E. coli-expressed human bitterness receptor hT2R4 and an indium tin oxide (ITO)-based electrolyte-semiconductor		Denatonium	50 nM	Inexpensive, robust, and simple bitterness evaluation	Complex bioengineering process of E. coli bacteria
	[118]	Bitterness receptor T2R4 immobilized on QCM transducer		Denatonium	5 nM	High sensitivity and selectivity, high efficiency in receptor expression and in situ purification	Complex and expensive fabrication process
	[127]	Taste bud cells on LAPS chip		Denatonium and ATP	0.1 nM for ATP	Both extracellular membrane potential changes and ATP release from a single taste bud cell can be recorded; good selectivity and stability	Complex cell-culturing process and short working time
	[109]	Rat cardiomyocytes cultured on MEA sensor		Denatonium benzoate and diphenidol	3.46 μM 2.92 μM	High sensitivity and selectivity for bitter and umami tastants	Complex fabrication process and short working time
	[128]	Coculturing Caco-2 cells and SH-SY5Y cells on CIS chip		Phenylthiocarbamide (PTC), propylthiouracil (PROP), salicin, and difenidol	100 μM 100 μM 400 μM 100 μM	Cell coculturing enriched bitter receptors on sensor	Complex cell-culturing process and professional operation
	[129]	Coculturing taste and neuronal cells		Denatonium benzoate	5.0 mM	Intercellular signal communication, good selectivity, and fast response time	Complex cell-culturing process and expensive instruments to record results
	[130]	Coculturing taste and neuronal cells on polystyrene cell culture plates coated with a decellularized tongue extracellular matrix		Saccharin	100 μM	Ensures the functional taste cell-specific phenotypes and improves the adhesion and sensitivity of taste cells; visible bitterness detection	Complex fabrication and cell-culturing process
Other bitter taste sensors	[139]	Interdigitated electrodes (IDEs) on a polymer chip prepared by laser-induced graphene technique		L-Tryptophan	5.205 μM	Fast, low-cost, and simple-to-operate	Poor selectivity to discriminate tastants

## Data Availability

Not applicable.
